# The Promising Therapeutic Potential of Celastrol for Fibrotic Diseases: A Systematic Literature Review on Its Mechanism

**DOI:** 10.7759/cureus.44269

**Published:** 2023-08-28

**Authors:** Nurin Yasmin Mohd Khairudin, Nasibah Azme, Nurdiyana Nasrudin, Siti Aznida Ab Karim

**Affiliations:** 1 Faculty of Medicine, Universiti Teknologi MARA, Shah Alam, MYS

**Keywords:** in vitro, in vivo, mechanism of action, fibrotic diseases, celastrol

## Abstract

Celastrol is a pentacyclic tripterine sourced from *Tripterygium wilfordii* hook root. Celastrol can exert certain biological functions such as antitumor, anti-inflammatory, and antiproliferative properties. Celastrol was shown from the previous literature to be capable of attenuating many fibrotic diseases. As the effects of various fibrotic diseases such as atherosclerosis, cancer, and ischemia affect more people with devastating repercussions, this warrants celastrol to be exploited as a phytotherapeutic drug. The purpose of this study is to review previous research and identify the proposed therapeutic mechanisms of celastrol in fibrotic diseases focusing on both the *in vitro* and *in vivo* experimental models.

A systematic literature search on Web of Science (WoS), Scopus, and ScienceDirect that included articles published between 2012 and 2022 was carried out using the keywords “celastrol”, “tripterine”, “fibrotic disease”, and “fibrosis”. After screening the initial search yield of 405 articles, 25 articles were included in this review.

The study findings summarize the potential therapeutic mechanism of celastrol in the attenuation of fibrotic diseases in *in vivo* and *in vitro* experimental models. It shows that celastrol is useful as a treatment means. However, more studies are needed on the effects of celastrol on humans to carry out clinical trials to verify the long-term benefits of celastrol.

## Introduction and background

Introduction

Fibrosis is often the result of the chronic progression of inflammatory diseases, where the wound-healing mechanism becomes dysregulated. This phenomenon is irreversible, and as a result, fibrous connective tissues are expressed in excess, leading to accumulation [1]. One of the major components in the development of fibrosis is myofibroblasts [2]. These cells contribute to fibrosis, which results in excess synthesis, deposition, and remodeling of the extracellular matrix [3]. The progression of fibrotic diseases leads to damaged or inflamed tissue, organ malfunction, and eventually death, as fibrosis can affect every tissue in the body [1,4-6].

Current treatments for fibrotic diseases include FDA-approved anti-fibrotic drugs such as nintedanib and pirfenidone. However, there is rising interest in phytotherapeutics, including celastrol, which has been proven to play a role in the attenuation of fibrosis. Celastrol, also known as tripterine, is a quinine methide triterpenoid that is the primary active component extracted from the root of *Trypterigium wilfordii*. *Trypterigium wilfordii*, also known as Thunder of God Vine, is a perennial vine native to the continent of Asia [7].

Multiple studies have reported that celastrol can activate pathways to counter autoimmune, tumor, neurodegenerative, oxidant, and most importantly, inflammatory diseases [8-13].

In this review, the different mechanisms exerted by celastrol on various fibrotic diseases, both in vivo and in vitro, will be examined. This review will also discuss fibrotic diseases along with their respective mechanisms.

Objective

The aim of this study was to systematically review the mechanisms by which celastrol minimizes the effects of fibrotic diseases in vivo and in vitro.

Methodology

The review was conducted in accordance with the reporting standards recommended in the Preferred Reporting Items for Systematic Reviews and Meta-Analyses (PRISMA) statement.

Formulating Questions

To guide the review, we identified the types of evidence required to answer the research questions. We employed the Domain, Determinant, and Outcome (DDO) format to obtain relevant answers. The search strategy was as follows:

Domain - "fibrotic disease" OR "fibrosis"

Determinant - "celastrol" OR "tripterine"

Outcome - "mechanism" OR "benefits" OR "cause".

Comprehensive Literature Search

The search procedure was conducted using bibliographic databases, namely Web of Science (WoS), Scopus, and ScienceDirect, to address the review question. The comprehensive literature search involved evaluating the eligibility of articles, crafting a search strategy for the identification of studies, selecting studies, and extracting data.

Eligibility Criteria

The inclusion and exclusion criteria were established among the authors before initiating the review process. The inclusion criteria for this study were research articles focusing on the effects of celastrol or tripterine on fibrotic diseases or fibrosis. The exclusion criteria were studies related to non-fibrotic diseases. Studies that did not meet the specific inclusion criteria were excluded.

Search Strategy for Identification of Studies

The published studies were carefully searched and identified. The electronic databases were searched using the following query: ("celastrol" OR "tripterine") AND "fibrotic disease" OR "fibrosis".

Published Articles

The term "published articles" refers to any articles that have appeared in peer-reviewed journals. For this study, published articles were located using computer-based information searches. The references in the selected articles were manually analyzed by all four researchers to identify additional articles for inclusion in this review.

Screening of Titles and Abstracts

The relevance of each article was determined by screening its title and abstract according to a coding study guide. This screening was performed by two authors (N.Y.M.K. and N.A.). In cases of disagreement about an article, the third and fourth authors (N.N. and S.A.A.K.) were consulted for resolution. Once all researchers reached a consensus on suitable titles and abstracts, those articles were earmarked for full-text retrieval. Relevant articles were downloaded into Mendeley Reference Manager software. Any articles that did not meet the criteria were excluded from the study. Prior to this, duplicate articles that had been downloaded were identified and removed.

Obtaining and Selecting Full-Text Published Articles

Full-text articles were obtained and downloaded from the selected databases. Articles without full text available were excluded from the study. The subsequent selection of suitable full-text articles was carried out by N.A. and N.N., who are experts in both methodology and the topic under review. Only original research articles were included in this study.

Critical Appraisal

All four independent researchers performed critical appraisals to assess the quality of the articles and the appropriateness of their study design in relation to the research objective. The critical appraisal was guided by the Consolidated Standards of Reporting Trials (CONSORT) checklist for cross-sectional studies. Articles that failed to meet the objectives or were of poor quality were excluded and discussed in greater detail.

Data extraction

Data extraction was carried out by two independent researchers to ensure inter-rater reliability and to minimize data entry errors. The context of the published articles was incorporated into a spreadsheet, which described each study and its subjects. Key findings from the studies were collated into a standardized data extraction form. A comprehensive list of the included studies was subsequently generated.

Study Flowchart

**Figure 1:**
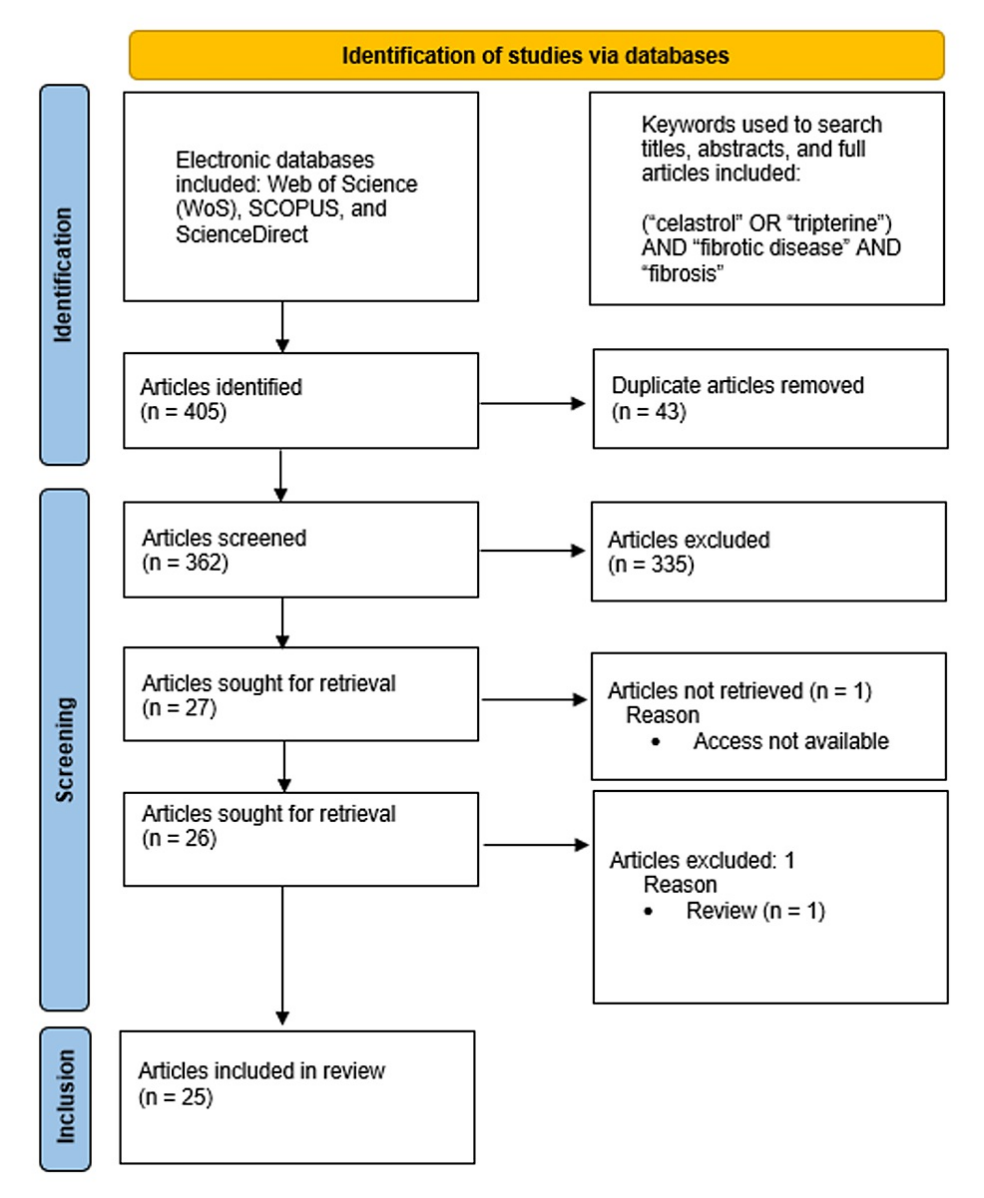
Study Flowchart

The workflow for the search and selection of articles conducted in this study is illustrated in Figure 1.

Data Management

All relevant articles were manually coded in spreadsheets. The electronic spreadsheets were utilized to import the data into Excel sheets (Microsoft Corporation, Redmond, Washington, United States) for data analysis.

## Review

Results 

The initial search yielded a total of 405 articles. Of these, 43 were removed due to duplication. The titles of the remaining 362 articles were screened, resulting in the exclusion of 335 studies as they were found to be irrelevant to the scope, question, and objectives of our review. After screening 26 articles, one was excluded because it was a review paper.

A total of 25 articles met the inclusion and exclusion criteria and were systematically reviewed. The shortlisted articles used celastrol as a form of treatment or intervention. However, the mechanisms through which celastrol functioned varied among the studies. Of these, 10 studies (40%) utilized animal research models (in vivo), two studies (8%) employed in vitro methods, and 13 studies (52%) used both in vivo and in vitro approaches. A more detailed breakdown of the findings can be found in Tables 1, 2.

**Table 1:** Celastrol Attenuation Route(s) and Mechanisms in an In Vivo Model STAT3: signal transducer and activator of transcription 3; MAPK: mitogen-activated protein kinases; ERK: extracellular signal-regulated kinases; HSF1: heat shock factor 1; HO-1: heme oxygenase 1; LAD: left anterior descending coronary artery; PI3K: phosphatidylinositol-3-kinase; Akt: protein kinase B; NOX2: nicotinamide adenine dinucleotide phosphate hydrogen (NADPH) oxidase-2; GSK3B: glycogen synthase kinase 3 beta; AVICs: aortic valvular interstitial cells; CCl4: carbon tetrachloride; AMPK: AMP-activated protein kinase 1; SIRT3: nicotinamide adenine dinucleotides (NAD)-dependent deacetylase sirtuin-3, mitochondrial; TGF-β1: transforming growth factor-beta1; Smad; suppressor of mothers against decapentaplegic; DEN: diethylnitrosamine; CLP: cecal ligation and puncture; NF-κB: nuclear factor kappa light chain enhancer of activated B cells; C57BL/6: C57 black 6;  PRDXs: peroxiredoxins; BLM: bleomycin;   Nrf2: nuclear factor erythroid 2-related factor; Keap1: kelch-like ECH-associated protein; mTOR: mammalian target of rapamycin; EMT: epithelial-mesenchymal transition; PAB: pulmonary artery banding; Pkd1: polycystic kidney 1; miR: miRNA; TG: transgenic; UUO: unilateral urethral obstruction; HMOX-1: heme oxygenase 1; CXCR4: C-X-C motif chemokine receptor 4; MMP-2: matrix metalloproteinase-2; MMP-9: matrix metalloproteinase-9.

Study	In vivo model	Fibrotic disease	Celastrol treatment	Exposure	Mechanism(s) involved
Ye et al. 2020 [14]	C57BL/6 male mice	Cardiac fibrosis	1 mg/kg/2 d	Transverse aortic constriction	STAT3 activity activation
Cheng et al. 2016 [15]	Kunming mice	Cardiac fibrosis	1 mg/ kg i.p. daily	Transverse aortic constriction	MAPK/ERK signaling inhibition
Der Sarkissian et al. 2014 [16]	Sprague-Dawley rats	Ischemic myocardium	1 mg/kg	Coronary artery occlusion	HSF1/HO-1 pathways activation
Der Sarkissian, et al. 2014 [17]	Female Sprague-Dawley rats	Ischemic myocardium	1 mg/ kg i.p. daily for 14 days	Permanent occlusion	HO-1 activation
Tong et al. 2018 [18]	Male Sprague-Dawley rats	Myocardial ischemia/reperfusion	2–6 mg/kg	LAD occlusion and a 4-h reperfusion-induced	PI3K/Akt pathway activation
Liu et al. 2020 [19]	Male New Zealand white rabbits	Aortic valve calcification	1 mg/kg/day	0.5% cholesterol-enriched chow plus 25,000 IU/day of vitamin D2	Inhibition of NOX2-mediated GSK3B/b-catenin pathway in AVICs
Wang et al. 2020 [20]	Male Sprague-Dawley rats	Liver fibrosis	0.25–1 mg/kg	CCl_4_-induced	AMPK‐SIRT3 signaling activation
Jiang et al. 2019 [21]	Wistar rats	Liver	4, 8 mg/kg/ body weight	CCl_4_ induced	TGF-β1/Smad inhibition
He et al. 2013 [22]	Rats	Liver fibrosis	2, 4, 8 mg/kg	DEN	TGF-β1 inhibition
El-tanbouly et al. 2017 [23]	Male Sprague-Dawley rats	Hepatic dysfunction	1 mg/kg, i.p.	CLP-induced	Attenuate NF-κB activation
Luo et al. 2022 [24]	C57BL/6 male mice	Hepatic fibrosis	0.25–1.0 mg/kg	CCl_4_-induced	PRDXs inhibition and HO-1 signaling activation
Divya et al. 2016 [25]	Male Wistar albino rats	Pulmonary	1, 2, 5, 7, 10 mg/kg/ body weight	BLM-induced	Nrf2/ Keap1 activation
Divya et al. 2017 [26]	Male Wistar albino rats	Pulmonary fibrosis	5 mg/kg/ body weight	BLM-induced	Inhibition of PI3K/Akt mediated mTOR expression
Divya et al. 2018 [27]	Male Wistar albino rats	Pulmonary fibrosis	5 mg/kg/ body weight	BLM-induced	EMT inhibition
Kurosawa et al. 2021 [28]	C57BL/6 mice	Pulmonary arterial hypertension and right ventricular failure	1 mg/kg/48 h	PAB Surgery	NF-κB inhibition
Li et al. 2022 [29]	Male Sprague-Dawley rats	Pulmonary fibrosis	1 mg/kg for 4 weeks	Monocrotaline	NF-κB signaling pathway inhibition
Tang et al. 2018 [30]	Male BALB/C mice	Renal fibrosis	1 mg/kg daily for 7 days	UUO- induced	Activation of Cannabinoid receptor 2 expression
Chang et al. 2018 [31]	Pkd1 miR TG mice with a C57BL/6 background	Polycystic kidney	1 or 2 mg/kg/day	Hypomorphic mutation of mice	AMPK activation
Yang et al. 2021 [32]	Male C57BL/6 mice	Vascular calcification in chronic kidney disease	1 mg/kg	5/6 nephrectomy	Upregulation of HMOX-1 activation
Kun-Ming et al. 2020 [33]	Mice	Hepatocellular carcinoma	2 mg/kg	Hepatocellular carcinoma cell implantation	CXCR4-related signal pathway inhibition
Chang et al. 2016 [34]	Male Sprague-Dawley rats	Hepatocellular carcinoma	2, 4, 8 mg/kg/day	DEN-induced	Inhibition of MMP-2 and MMP-9 expression
Jiang et al. 2020 [35]	Female BALB/C mice	Systemic sclerosis	1 mg/kg	BLM-induced	Activation of cannabinoid receptor 2 expression

**Table 2:** Celastrol Attenuation Route(s) and Mechanisms in an In Vitro Model HUVEC-12: human umbilical vein endothelial cells; EndMT: endothelial-mesenchymal transition; LEC: lens epithelial cell; TGF-β2: transforming growth factor-beta2; MiR-21: microRNA-21; HK-2: human kidney 2; Ang II: angiotensin II; LX-2: human hepatic stellate cells; HSC: hepatic stellar cells; HCC: hepatocellular carcinoma; VSMC: vascular smooth muscle cells.

Study	Type of cell lines	Fibrotic disease	Celastrol treatment	Exposure	Mechanism(s) involved
Gong et al. 2017 [8]	HUVEC- 12 cells	Cardiac fibrosis	200 nmol/L	TGF-β1 induced	inhibition of TGF-β1-induced EndMT through TGF-β1/Smads signaling pathway
Ye et al. 2020 [14]	Rat primary cardiomyocytes and H9C2	Cardiac fibrosis	0.25, 0.5, 1 nmol/L	Ang II	STAT3 activation
Cheng et al. 2016 [15]	Cardiac fibroblasts	Cardiac fibrosis	0.2, 1, 5 μM	Fibroblast cells	miR-21/ERK signaling pathway inhibition
Der Sarkissian et al. 2014 [17]	Rat cardiomyoblast H9c2 cells	Ischemic myocardium	10^−10^–10^−6^ M	Hypoxia	HO-1 activation
Liu et al. 2020 [19]	Primary porcine AVICs	Aortic valve calcification	10 nmol/L	Infected with β-gal virus	inhibition of NOX2-mediated GSKB/b-catenin pathway in AVICs
Wang et al. 2020 [20]	Primary rat HSCs isolated from male Sprague‐Dawley rats	Liver fibrosis	10, 20, 40 µmol/L	HSC activation	AMPK‐SIRT3 signaling activation
Luo et al. 2022 [24]	LX-2 and mouse hepatic stellate cell	Liver fibrosis	0.5–1.0 µmol/L	HSC activation	PRDXs inhibition and HO-1 signaling activation
Divya et al. 2018 [27]	Human alveolar epithelial adenocarcinoma A549 cells	Pulmonary fibrosis	5 µM/ml	TGF-β	inhibition of TGF-β/Smad signaling induced EMT
Kurosawa et al. 2021 [28]	Human lung sample - Small pulmonary arteries of humans - Neonatal rat cardiomyocytes	Pulmonary arterial hypertension	1 μmol/L	Hypoxia	NF-κB inhibition
Tang et al. 2018 [31]	HK-2 cells	Renal fibrosis	0 to 500 nM	TGF-β1	Activation of cannabinoid receptor 2 expression
Yang et al. 2021 [32]	VSMCs from Sprague Dawley rats	Vascular calcification in chronic kidney disease	1, 10, 20, 50 nM	Calcifying medium: 10 mM β-glycerophosphate	Upregulation of HMOX-1 activation
Kun-Ming et al. 2020 [33]	Human HCC: HepG2 and Hepa3B cell lines	Hepatocellular carcinoma	0.1–1.0 mmol	N/A	CXCR4-related signal pathway inhibition
Wang et al. 2019 [36]	LEC line SRA01/04	Lens epithelial cell fibrosis	0.1–1.0 mmol	TGF-β2	Inhibition of TGF-β/Smad and Jagged/Notch signaling pathways activation-induced EMT

Fibrotic Diseases

The studies on fibrotic diseases included in this review focused on fibrotic progression related to cardiac, liver, pulmonary, renal, and cancer conditions, as well as systemic sclerosis and lens epithelial cell fibrosis. Based on the reviewed studies across three databases, the most prevalent fibrotic diseases treated with celastrol were cardiac-related diseases, with seven studies (28%), followed by liver-related diseases with six studies (24%), pulmonary-related diseases with five studies (20%), renal-related diseases with three studies (12%), cancer-related diseases with two studies (8%), systemic sclerosis with one study (4%), and lens epithelial cell fibrosis also with one study (4%).

Study Models

Study models in the selected studies can be categorized into two types: animal models and cell models. For rat animal model studies, two strains were commonly used: Sprague Dawley rats and Wistar albino rats. Mice, on the other hand, showed a prevalence in the strains of C57BL/6, BALB/C, Pkd1 miRNA transgenic mice, and Kunming mice, listed in ascending order. One study modeled aortic valve calcification on male New Zealand white rabbits.

The use of cells was more varied compared to animal models, as different fibrotic diseases required different triggers and cell conditions. Cells were also used to mimic the closest pathogenesis in human cells. The cells used included HUVEC-12, human LEC line SRA01/04, HSC, cardiac fibroblasts, HK-2, rat primary cardiomyocytes H9C2 cells, primary porcine AVICs, human alveolar epithelial adenocarcinoma A549 cells, LX-2, mouse hepatic stellate cells, primary rat HSCs, human HCC lines HepG2 and Hepa3B, human lung samples, small pulmonary arteries of humans, neonatal rat cardiomyocytes, and VSMCs.

Mechanisms

Referring to both Table 1 and Table 2, there are overlapping mechanisms across these 25 studies. The most prevalent mechanism is AMPK signaling activation, which appeared in three studies. This is followed by TGF-β inhibition, NF-κB signaling pathway inhibition, activation of cannabinoid receptor 2 expression, PI3K/Akt pathway activation, HO-1 activation, EMT inhibition, Nox2 pathway inhibition, MMP-2 pathway inhibition, MMP-9 pathway inhibition, HMOX-1 activation, Nrf2/Keap1 activation, STAT3 activation, miR-21/ERK inhibition, and p53 activation. The studies showed that celastrol operates through multiple pathways, reinforcing its potential as a phytotherapeutic drug. As all the reviewed studies exhibited a certain extent of positive results, the working mechanisms through which celastrol functions will be further elucidated in the next section.

Discussion

This section focuses on and summarizes the mechanisms by which celastrol is used to attenuate various types of fibrotic diseases.

Cardiac-Related Fibrotic Disease

Seven studies used celastrol as a means to alleviate cardiac-related diseases. These diseases consist of cardiac fibrosis (CF) and dysfunction, cardiomyocyte hypertrophy, aortic valve calcification, and ischemic myocardium. The pathways included are as follows: PI3K/Akt pathway activation, HSF1/HO-1 pathways, HO-1 expression, inhibition of the Nox2-mediated GSK3B/b-catenin pathway in AVICs, STAT3, TGF-β1-induced EndMT through TGF-β1/Smads signaling pathway, and MiR-21/ERK signaling.

A study investigating cardiac dysfunction found STAT3 to be involved in the beneficial effects of celastrol. The cardiac dysfunction induced by angiotensin II was inhibited by celastrol via STAT3 inhibition [14]. Another study looked into the mechanism involved in both cardiac fibrosis and dysfunction. These were found to be attenuated by celastrol via the MiR-21/ERK signaling pathway [15].

The upregulation of miR-21 is a result of pressure overload in the transverse aortic constriction (TAC) model, leading to the progression of fibrosis. The overexpression of miR-21 was found to trigger ERK/MAPK activation. The TAC model was also tested to undergo an upregulation in ERK1/2 phosphorylation via western blotting. Upon treatment using celastrol, both the miR-21 and ERK1/2 phosphorylation significantly decreased. A key molecule in attenuating CF was TGF-β1. The in vitro part of this study yielded a positive result, with celastrol found to inhibit TGF-β1/miR-21 signaling [15].

Another study that made use of TGF-β1 signaling was conducted by Gong et al. [8]. The HUVEC-12 cells were incubated in TGF-β1 as a fibrosis trigger and followed by celastrol as a treatment. Upon exposure to TGF-β1, mRNA levels of collagen I, III, smooth muscle alpha-actin (α-SMA), and fibronectin were found to significantly increase, and the CD31 marker was found to decrease. Incubation with celastrol led to a blocking of the EndMT pathway induced by TGF-β1, indicated by a decreased expression of mesenchymal markers.

Another mechanism of interest involves the HO-1 and HSF1/HO-1 pathways [16,17]. These studies investigated the role of HO-1 expression in ischemic myocardium attenuation. Celastrol treatment in the study by Der Sarkissian et al. [16] was found to reduce cardiac injury by activating the heat shock response (HSR) and upregulating HO-1. A follow-up study indicated that the involved HSR was HSF1. Celastrol promotes cell survival through HSF1 activation coupled with HO-1 upregulation, thus exhibiting anti-fibrotic effects [17].

Aside from the HO-1 pathway being exploited for attenuating fibrotic progression in myocardial ischemia, the PI3K/Akt pathway is another useful pathway [18]. Celastrol reduces the high mobility group box 1 (HMGB1) protein signal, which is induced by I/R in Sprague-Dawley rats. This HMGB1 signal reduction is achieved via PI3K/Akt pathway upregulation by celastrol. Furthermore, when treated with the PI3K inhibitor LY294002, the anti-fibrotic, anti-inflammatory, and anti-oxidative effects of celastrol were reversed.

Another cardiac-related fibrotic disease benefiting from celastrol treatment is aortic valve calcification. Simulated calcification in AVIC cells resulted in NOX2 upregulation, which was significantly diminished by celastrol treatment [19]. This positive result is due to the activation of NOX2-mediated GSK3B/b-catenin signaling, promoting fibrogenic and osteogenic responses in AVICs. By decreasing NOX2 expression, GSK3B/b-catenin signaling is blocked, resulting in the reduction of fibrotic and calcific markers.

Liver-Related Fibrotic Disease

Six studies employed celastrol in treating liver-related fibrotic diseases. The mechanisms investigated include AMPK‐SIRT3 signaling, TGF-β1, TGF-β1/Smad, targeting PRDXs, and HO-1 induction to promote ferroptosis of activated-HSCs, and NF-κB activation.

In liver fibrosis, celastrol treatment suppressed inflammation both in vivo and in vitro via AMPK‐SIRT3 signaling [20]. The drug elevated SIRT3 and AMPK expression in activated HSCs and fibrotic rat livers, activating AMPK-SIRT3 signaling and diminishing pro-inflammatory factors like IL-1β, IL‐6, IL‐18, TNF‐α, and IFN‐γ. Therefore, celastrol manifests anti-inflammatory properties against liver fibrosis.

In addition to AMPK-SIRT3, the TGF-β1 pathway contributes to celastrol's anti-fibrotic efficacy [21,22]. In a CCl4-induced liver fibrosis model, Jiang et al. [21] found that celastrol substantially reduced levels of hyaluronic acid (HA), laminin (LN), type IV collagen (Col IV), and procollagen III (PCIII). He et al. [22] also showed that celastrol alleviated liver damage and symptoms of liver fibrosis.

To further prove the anti-inflammatory effects of celastrol, NF-κB was inhibited to attenuate hepatic dysfunction [23]. The reduction in hepatic mRNA expression of NFκB, toll-like receptor 4 (TLR-4), and 5-lipoxygenase (5-LOX) mediated the anti-inflammatory effects of celastrol, coupled with the inhibition of NF-κB/p65 expression in the nucleus. In addition, mRNA expression of IL-6 decreased, causing the serum level to respond by reduction.

Besides inhibiting the inflammatory pathway, celastrol can also trigger ferroptosis of activated HSCs by targeting PRDXs and HO-1 expression [24]. Celastrol can upregulate the expression of HO-1, which leads to the build-up of lipid peroxidation (LPO), reactive oxygen species (ROS), and ferrum 2^+ ^(Fe2^+^), which triggers ferroptosis in the activated HSCs. As ferroptosis is triggered, regulated use of this programmed cell death can inhibit further progression of fibrotic cells, hence celastrol exhibits anti-fibrotic properties in liver fibrosis.

Pulmonary-Related Fibrotic Disease

Studies related to BLM-induced pulmonary fibrosis with celastrol as a treatment exploit the pathways involving Nrf2/Keap1- and PI3K/Akt-mediated mTOR expressions [25,26]. The Nrf2 activation by celastrol via Keap1-induced proteolysis was found to be favorable in terms of inhibition of inflammation and collagen accumulation as well as enhancement of antioxidant levels [25]. A follow-up study conducted by Divya et al. [26] investigated the role of PI3K/Akt-mediated mTOR expression in inducing autophagy. In the BLM-induced mice, the mTOR and PI3K/Akt expression was enhanced. Therefore, the inhibition of PI3K/Akt inhibited mTOR as PI3K/Akt was an upstream modulator of mTOR. With the inhibition of mTOR, autophagosomes can be formed to carry out autophagy, hence presenting the anti-fibrotic effects of celastrol on pulmonary fibrosis.

Divya et al. [27] further explored the mechanisms involved in celastrol attenuation of BLM-induced pulmonary fibrosis. The mechanism of interest was EMT mediated by growth Factor-b/Smad. In the BLM-induced rats, the epithelial markers were found to be decreased while mesenchymal markers increased, thus indicating the development of fibrosis. A celastrol treatment reversed these cellular changes via heat-shock protein 90 (HSP90) inhibition. Taken together, it is evident that celastrol can attenuate BLM-induced pulmonary fibrosis.

Next, the NF-κB signaling pathway also seems to be a pathway of interest when it comes to a celastrol treatment of pulmonary-related disease, namely, pulmonary arterial hypertension [28,29]. There has been evidence of celastrol attenuating both in vivo and in vitro models via NF-κB. In an animal model, celastrol reduced the infiltration of macrophage, decreased the levels of pro-inflammatory cytokine levels, increased the level of anti-inflammatory cytokine, and inhibited the NF-κB signaling pathway. In in vitro models, celastrol suppressed the proliferation of human pulmonary artery smooth cells under hypoxia, which blocked the progression of fibrosis as well as inhibited Basigin (Bsg) and Cyclophilin A (CyPA), which reduced NF-κB, inflammatory cytokines, and ROS levels [28]. Inhibition of Bsg and CyPA is significant as binding of these two compounds promotes the release of inflammatory cytokines [28]. Therefore, the anti-inflammatory properties of celastrol remain evident.

Renal-Related Fibrotic Disease

The studies involving celastrol-treated renal-related fibrotic diseases include renal fibrosis, polycystic kidney disease, and vascular calcification in chronic kidney disease. Celastrol attenuates these diseases via cannabinoid receptor 2 (CB2) expression, AMPK, and HMOX-1, respectively [30-32].

In terms of cannabinoid receptor 2 expression, UUO- or TGF-β1-induced renal fibrosis can be attenuated via upregulation of CB2. This is due to CB2 downregulation in renal fibrosis being a product of TGF-β1 expression. As TGF-β1 levels increase, the pro-fibrotic factor Smad3 is triggered. Upon treatment with celastrol, the Smad3 activation is inhibited, therefore allowing upregulation of CB2, which is an anti-fibrotic factor [30].

To further exploit the benefits of celastrol, its use on polycystic kidney disease (PKD) and vascular calcification in chronic kidney disease demonstrates its anti-inflammatory effects. The mechanisms of AMPK signaling and HMOX-1 are involved in the alleviation process. The AMPK signaling functions by the upregulation of AMPK by celastrol. It is found that the enhanced phosphorylation of AMPK can retard the cystogenesis of renal cells and disrupt the progression of PKD to a certain extent [31]. As for HMOX-1, celastrol has been found to enhance the expression of HMOX-1. This in turn represses the oxidative stress markers and thus blocks the calcification of the vascular [32].

Cancer-Related Fibrotic Disease

Both studies of celastrol-treated cancer explored HCC via the expression of CXCR4, p53 activation, and MMP expression, specifically MMP-2 and MMP-9. Celastrol was found to reduce the expression of CXCR4, which caused the downstream pathway PI3K/Akt to be reduced as CXCR4, which acted as a precursor to the pathway. CXCR4 signaling pathway blockage was found to be beneficial in tumor suppression as CXCR4 contributed to tumor progression, angiogenesis, and metastasis [33]. 

Next, MMP-2 and MMP-9 expression as well as p53 activation were also found to contribute to DEN-induced HCC alleviation [34]. The anti-tumor effects of celastrol were attenuated via HCC cell apoptosis due to the accumulation of p53. Celastrol was also found to block the metastasis of HCC via MMP-2 and MMP-9 reduction. These multiple pathways further strengthened the functionality of celastrol in fibrotic diseases, especially cancer.

Systemic Sclerosis

Celastrol exerts its anti-fibrotic effects on systemic sclerosis, which leads to a reduction in inflammatory conditions via CB2 signaling pathway. Celastrol can act as a CB2 agonist, which means it can trigger CB2 activation. The activation is significant in reducing TNF-α, inducible nitric oxide synthase (iNOS), IL-1β, and C-X-C motif chemokine ligand 10 (CXCL10) expression that interplays in chronic inflammatory response [35].

Lens Epithelial Cell Fibrosis

Another fibrotic disease that utilizes EMT via TGF-β/Smad as a means of attenuation is LEC fibrosis. TGF-β2-induced Smad2/3 phosphorylation in LECs has been found to be reduced by celastrol. Inactivation of TGF-β2 leads to EMT inactivation in which celastrol reduces the expression of fibronectin, Col IV, and α-SMA, thus causing the migration and proliferation of LECs to be inhibited as well as causing the arrest of the cell cycle [36]. 

## Conclusions

Currently, celastrol shows promising anti-fibrotic effects against various fibrotic diseases, demonstrating both anti-inflammatory and anti-tumor properties. Given that multiple therapeutic targets and both direct and indirect mechanistic pathways have been identified in vitro and in vivo, this suggests that celastrol holds significant therapeutic potential for treating fibrotic diseases. However, there is a lack of studies exploring its toxicity threshold, which could lead to undesirable side effects. Further investigations of this treatment should include testing in various in vivo animal models and in vitro cell models, as well as across different types of fibrotic diseases, before advancing to clinical trials. This comprehensive approach will help to better assess celastrol's viability as a promising therapeutic drug.
